# Innovative perspectives in limbic surgery using deep brain stimulation

**DOI:** 10.3389/fnins.2023.1167244

**Published:** 2023-05-18

**Authors:** José Damián Carrillo-Ruiz, José Rodrigo Carrillo-Márquez, Jesús Quetzalcóatl Beltrán, Fiacro Jiménez-Ponce, Luis García-Muñoz, José Luis Navarro-Olvera, René Márquez-Franco, Francisco Velasco

**Affiliations:** ^1^Stereotactic, Functional & Radiosurgery Unit of Neurosurgery Service, Mexico General Hospital, Mexico City, Mexico; ^2^Research Direction, Mexico General Hospital, Mexico City, Mexico; ^3^Neuroscience Coordination, Psychology Faculty, Anahuac University, Mexico City, Mexico; ^4^Faculty of Health Sciences, Anahuac University, Mexico City, Mexico; ^5^Alpha Health Sciences Leadership Program, Anahuac University, Mexico City, Mexico

**Keywords:** limbic surgery, psychosurgery, aggressiveness, anxiety, drug usage, eating disorders, dementia, transhumanism

## Abstract

Limbic surgery is one of the most attractive and retaken fields of functional neurosurgery in the last two decades. Psychiatric surgery emerged from the incipient work of Moniz and Lima lesioning the prefrontal cortex in agitated patients. Since the onset of stereotactic and functional neurosurgery with Spiegel and Wycis, the treatment of mental diseases gave attention to refractory illnesses mainly with the use of thalamotomies. Neurosis and some psychotic symptoms were treated by them. Several indications when lesioning the brain were included: obsessive-compulsive disorder, depression, and aggressiveness among others with a diversity of targets. The indiscriminately use of anatomical sites without enough scientific evidence, and uncertainly defined criteria for selecting patients merged with a deficiency in ethical aspects, brought a lack of procedures for a long time: only select clinics allowed this surgery around the world from 1950 to the 1990s. In 1999, Nuttin et al. began a new chapter in limbic surgery with the use of Deep Brain Stimulation, based on the experience of pain, Parkinson’s disease, and epilepsy. The efforts were focused on different targets to treat depression and obsessive-compulsive disorders. Nevertheless, other diseases were added to use neuromodulation. The goal of this article is to show the new opportunities to treat neuropsychiatric diseases.

## Introduction

Psychiatric illnesses were treated at the beginning of the 20*^th^* century with brain surgery (Freeman and Watts, 1948). No drugs or psychotherapy existed at that time. It was until the 1940s–1950s that brain surgery and psychiatric drugs were utilized. Limbic surgery declined in the 1970s and only few clinics in the world performed these procedures. The arrival of deep brain stimulation (DBS) for neurological and psychiatric diseases brought amelioration of symptoms of obsessive-compulsive disorder (OCD) and depression (Nuttin et al., 1999). This has motivated the scientific community to retake limbic surgery as a discussion topic.

Theoretically, DBS could treat every mental disease, but is important to know the exact mechanism involved in each illness. Moreover, at this moment this proposal is not totally achievable, because of the lack of precision regarding neuroanatomy and neurophysiology of brain circuitries (i.e., connectome and synaptome) and neurotransmitters involved in each mental and neurological disease. After discovering the pathophysiology of every disease, it could be easier to treat specific illnesses and/or modify the nervous system to be more effective.

The criteria to perform psychosurgery includes: (1) The presence of brain nuclei lesions or stimulation in different animal models of mental illness (Oterdoom et al., 2020). (2) The surgical experiences of limbic surgery in patients with psychiatric diseases (Langevin, 2012; García-Muñoz et al., 2019). (3) DBS use in neurological illness to treat mental symptoms and modify them with neuromodulation; based on the experience learned from STN, Thalamus, or GPi in patients with Parkinson’s disease, pain, and epilepsy (Hosobuchi et al., 1973; Benabid et al., 1991; Lee et al., 2019). (4) DBS treatment in OCD and depression symptoms alleviation (Nuttin et al., 1999; Marquez-Franco et al., 2022).

The next lines demonstrated the new indications for DBS treatment in psychiatric pathologies: anxiety, eating disorders (EDs), substance use disorders, aggressiveness, schizophrenia, and Alzheimer’s disease (see Table 1).

**TABLE 1:** New indications of deep brain stimulation in psychosurgery.

References	Target	Disease	Results
Sturm et al., 2003	Nucleus accumbens	Anxiety/OCD	4 patients: 24–30 months with severity reduction
Luyten, 2020	Stria terminalis/bed ST		Good results in rats
Lipsman et al., 2017	Subcallosal cingulate	Anorexia	16 patients: 14 at 1 year; 57% with significant improvement
Liu et al., 2020	Nucleus accumbens		24 patients, 2 years; 36% improvement
Villalba-Martínez et al., 2020	SC + NA		8 patients; 6/12 yrs; > 10% improvement
De Salles et al., 2018	Anteromedial hypothalamus nucleus	Obesity	6 patients: protocol to develop
Kuhn et al., 2009	Nucleus accumbens (tobacco)	Drug addiction	3 patients: 30 months; 30% of cessation of smoke
Kuhn et al., 2014	Nucleus accumbens (opioids)		2 patients: 24 months; 100% of diminution of craving
De Ridder et al., 2016	Anterior cingulate cortex (alcohol)		1 patient 18 months free of intake alcohol
Kuhn et al., 2007	Nucleus accumbens (alcohol)		1 patient total remission of alcoholism
Franzini et al., 2013	Posterior hypothalamus	Aggressiveness	7 patients: 3-11 yrs;65% reduction in OAS scale
Torres et al., 2013	Posterior hypothalamus		6 patients:6-82 mos 47% improvement on ICAP scale
Micieli et al., 2017	Posterior hypothalamus		4 patients; recording sleep pattern
Blasco-García de Andoain et al., 2021	Posteromedial hypothalamus		1 patient with Weaver syndrome: Good affective improvement
Benedetti-Isaac et al., 2015	Posterior hypothalamus		5 patients: 2–48 months 65% OAS scale improvement
Benedetti-Isaac et al., 2021	Posterior hypothalamus		19 patients: 18 months >50% OAS scale improvement
Contreras-Lopez et al., 2021	Posteriomedial hypothalamus		4 patients: 12 months; 50% aggressive scale
Laxton et al., 2010	Fornix and hypothalamus	Alzheimer disease	6 patients: 12 months “possible improvement”
Fontaine et al., 2013	Fornix and hypothalamus		1 patient: 12 months; “memory scales stabilized”
Lozano et al., 2016	Fornix and hypothalamus		42 patients: 12 months; no cognitive differences group <5 years to > 65 years
Leoutsakos et al., 2018	Fornix and hypothalamus		42 patients: 12 months: complete safety and security
Germann et al., 2021	Fornix and hypothalamus		39 patients: anatomical study; 72% accuracy
Targum et al., 2021	Fornix and hypothalamus		42 patients: 12 months: age analysis in young patients
Barcia et al., 2022	Fornix and hypothalamus		1 patient: 24 months; mild to moderate improvement
Ríos et al., 2022	Fornix and hypothalamus		46 patients: optimal electrodes positions lateral-posterior column of fornix
Kuhn et al., 2015	Nucleus basalis meynert	Alzheimer disease	6 patients: 4 responders; 11 mos: low improvement, safe intervention
Baldermann et al., 2018			10 patients: MRI analysis: Better improvement gross cortex
Dürschmid et al., 2020			2 patients: attenuated early EEG components
Jiang et al., 2022			8 patients: 12 mos; improve short memory-performance
Corripio et al., 2016	Na and ACC	Schizophrenia	7 patients: 2/3 Na, 2/4 ACC; 58% and 37% Amelioration
Wang et al., 2020	Habenula		1 of 2 patients respond
Corripio et al., 2022	Central tegmental area and substantia nigra pars reticulata		To explore

It resumed the recent scientific literature for treating psychiatric illnesses, and the results found by the researchers in their studies. SC, Subcallosal cingulate; Na, Nucleus accumbens.

## Materials and methods

We followed the National Library of Medicine standard search protocol, using PubMed and Google Scholar metasearch engines which are free and public. The search was conducted using Boolean Operators with the following Medical Subject Headings (MeSH): “psychosurgery,” “limbic surgery,” “deep brain stimulation,” “brain stimulation,” “stereotactic lesion,” “neuromodulation,” “depression,” “aggressiveness,” “drug usage,” “addiction,” “anxiety,” “eating disorders,” “electrodes,” “Alzheimer’s disease,” “Schizophrenia,” “transhumanism,” and “neuroethics.”

The inclusion criteria for the selected papers were: (1) new studies focused on limbic surgery in humans, and/or (2) animal studies that propose new targets for treating psychiatric illnesses, and (3) neuroethics regarding the implementation of new technologies for treating psychiatric pathologies. The exclusion criteria were: (1) articles written in another language than English, Spanish, or French and (2) articles that were not found as full-text articles. The results were synthesized and presented as a mini-review describing the new tendencies.

## Discussion

### Electrophysiology as a precursor to neuroscience and psychosurgery

Galvani’s work on animal electricity was a milestone in understanding the nature of the nerve impulse and led to the founding of electrophysiology (Piccolino, 1998; Bennett, 1999). This is focused on the clinical application for the management of various neurological diseases, and promoting laboratory research to expand the knowledge of biophysical neuronal functioning (Bennett, 1999; Feindel, 2007).

In the clinical field, this led to understanding the electrical stimulation of the nervous system. It helped to create more accurate diagnoses and treatments for neurological patients. Penfield, in the first half of the 20th century, mapped the topographic organization of motor and sensory homunculi in patients with epilepsy (Penfield and Boldrey, 1937). Later on, based on this experience Hassler et al. (1960), Benabid et al. (1991), Hosobuchi et al. (1973), Mundinger (1977), and other researchers developed DBS with chronically implanted devices for the management of various pathologies including Parkinson’s disease, essential tremor, pain, epilepsy, and neuropsychiatric disorders (Hosobuchi et al., 1973; Benabid et al., 1991; Lee et al., 2019; Marquez-Franco et al., 2022). More recently, responsive neurostimulation (RNS) has been used as a system that monitors brain waves and applies different voltages according to brain activity in a specific brain area (Albright et al., 2000).

Basic neuroscience research has helped to understand the cellular and molecular mechanisms of neural communication (Piccolino, 1998; Bennett, 1999). Emile du Bois-Reymond in the second half of the 20th century demonstrated the correlation between nervous tissue and bioelectricity. Researchers such as Dale, Loewi, Eccles, Katz, Miledi, Neher, Sakmann, and Kandel, unveiled the basic mechanisms involved in the transmission of information, processing, and neuronal communication (Albright et al., 2000). Hodgkin and Huxley described the biophysics of the electrical activity in neurons (Piccolino, 1998; Albright et al., 2000).

The convergence of basic and clinical neurophysiology in the last decades has created outstanding achievements. Undoubtedly, previous advances, like the development of dynamic and adaptable brain implants, are the prelude to developing correct DBS techniques and brain–machine interfaces (BMI) as a possibility for understanding the brain and treating illnesses (Hochberg et al., 2012; Raspopovic, 2021).

### Eating disorders (EDs)

Currently, EDs are a major health problem worldwide, and the reality is that humans must obtain energy and biochemical precursors from food (Hauck et al., 2020). In this way, in the feeding and nourishing mechanisms pleasure should be felt to assure survival. The activation of the reward system of dopamine circuitry (Thalamus Ventrolateral Tegmental nucleus) is involved in this process. The discovery of the activation of endocannabinoids and opioid peptides are some of the new contributions to functional neuroscience (Al Massadi et al., 2019). It has been proven in animal models that treating EDS with DBS can bring changes (Oterdoom et al., 2020). Therefore, surgeons should be very selective in the techniques implemented to assure a good outcome because it is translated to a better quality of life for patients. The last reports determined DBS is still an emerging treatment, yet human trials suggest two treatment possibilities: anorexia nervosa and obesity.

In the case of anorexia nervosa, DBS has been acknowledged as well as inserting electrodes in some key areas (Lipsman et al., 2013; Wu et al., 2013), including these anatomical targets: Rostromedial tegmental nucleus (Melse et al., 2016), Subcallosal cingulate (Sc) (Lipsman et al., 2017), Na (Nucleus accumbens) (Liu et al., 2020), and a combination of Sc + Na (Villalba-Martínez et al., 2020). Although promising results were published, further research should be made (Sobstyl et al., 2019; Treasure et al., 2020).

Regarding obesity, scientific evidence has determined some anatomical sites of the brain that could be modulated (Halpern et al., 2011; Torres et al., 2011; McClelland et al., 2013; Ho et al., 2015). After theoretical analysis, surgery was performed in humans by a few groups over the world. The possibility of treating EDs with anteromedial hypothalamus nucleus stimulation to promote the satiety effect is an option (De Salles et al., 2018). Another possible treatment was demonstrated by stimulating Na in a patient with obesity and depression using DBS to generate weight loss (Tronnier et al., 2018).

### Drug addiction (substance use disorders)

Is DBS an answer? Acknowledging and investigating dopaminergic reinforcement pathways must be discussed by neurosurgeons, neurologists, psychiatrists, and researchers to correctly treating these illnesses (Volkow et al., 2019). DBS has been utilized to treat substance addictions according to scientific literature (Chang et al., 2022).

In most animal models focused on drug usage, results tend to prove that the implementation of this technology helps to reduce addictions (Guercio et al., 2020). Although clinical trials provide encouraging results, the complex approach of creating human models has diminished in the last years (Wang et al., 2018). Moreover, Polyakov and Kholyavin (2022) reported crio-cyngulotomies as a possible safe treatment for psychological dependence in drug addiction.

Fattahi et al. (2022) described that opioid dependence can be treated by stimulating Na with high-frequency stimulation. Also, Kuhn et al. (2009, 2014) proposed treating opioid dependence with DBS in Na as well. DBS might be an answer to treat patients that require surgical intervention due to the failure of previous therapies and/or other treatments. Shallow to moderate addiction can be treated with psychiatric drugs, and intensive substance abuse opens the possibility to use neuromodulation and DBS.

Nowadays, tobacco addiction could be ameliorated after the stimulation of Na (Kuhn et al., 2009), and alcohol abuse disorder decreased with anterior cingulate cortex implantation (De Ridder et al., 2016), or with the use of Na stimulation (Kuhn et al., 2007). On the other hand, DBS in non-substance addictions has been poorly described, excluding EDs. This is a promising work based on results established by the scientific community (Volkow et al., 2019; Oterdoom et al., 2020).

Although research on substance use disorders is beginning, it is possible to get targets to treat addictions. Research in dependency problems is complicated because drug abuse can cause exclusion from studies (i.e., secondary pathologies and death) in subjects. The comorbidities can bring many mental disorders (e.g., OCD, depression, dementia), so it is important to create models of drug dependence in these subjects as well.

### Anxiety

Limbic surgery began to treat psychiatric patient’s suffering. The procedure consisted in lesioning the frontal lobe as proposed by Freeman and Watts (1948). It was possible to diminish all the symptoms of anxiety, but with unwished secondary effects (Lichterman et al., 2022). At that time, it existed neither consensus of psychiatrists nor the Diagnostic and Statistical Manual of Mental Disorders (DSM). After several decades, anxiety patients were treated with psychiatric drugs and/or therapy. Brain surgical procedures were practically abandoned and reduced to lesions of the following structures: anterior cingulate circumvolution (ACC), subcaudate nuclei, and anterior capsule (Leiphart and Valone, 2010; Patel et al., 2013). Nevertheless, Nuttin et al. (1999) referred to the possibility of neuromodulating the internal capsule to treat OCD. Some of these patients had anxiety alleviation.

Diverse authors reported benefits in treating OCD, and nervousness with DBS (Sturm et al., 2003; Patel et al., 2013; Velasques et al., 2014; Freire et al., 2020; Ganz, 2022). For anxiety, some targets have been reported lately, including Na (Sturm et al., 2003), Papez ring (Hescham et al., 2015), and the Bed Nucleus of Stria Terminalis (BNST) (Luyck and Luyten, 2015; Luyten, 2020). The results indicated an amelioration of OCD symptoms, but also a decrease in fretfulness and fear. Li et al. (2022) described that hypothalamic DBS can be used in brief periods to treat anxiety and arrest relapse in a mice model. Further basic and clinical research should be done to prove efficacy (Li et al., 2022).

### Aggressiveness

One of the most important indications to perform limbic surgery is the presence of aggressiveness in individuals with psychiatric illnesses. Symptoms by themselves are not a disorder. Brain lesioning surgery has been performed in patients with good results in diverse clinical studies (García-Muñoz et al., 2019; Marquez-Franco et al., 2022). The most frequent target used in surgery is the hypothalamus (Rizzi et al., 2017; Marquez-Franco et al., 2022), then the cingulum and amygdala (Langevin, 2012). These specific lesions brought good results, so the next step was to perform DBS.

Italians Franzini et al. (2013) and Rizzi et al. (2017) proposed the posterior hypothalamus to treat neuropathic pain, and it was transferred to patients with violent behavior with good outcomes. They were followed by the Spaniards with hypothalamic DBS (Torres et al., 2013, 2020; Blasco-García de Andoain et al., 2021), and by Colombian and Brazilian neurosurgeon groups with the same methods in clinical trials (Benedetti-Isaac et al., 2015, 2021; Micieli et al., 2017; Contreras-Lopez et al., 2021).

### Alzheimer’s disease and dementia

A very recent point is neuromodulation to treat amnesia and forgetting in patients with dementia and Alzheimer’s disease. The idea to stimulate sites where memory and learning is located is not new because murine models were made. After, Laxton et al. (2010) introduced electrodes in the limbic system, fornix and hypothalamus in individuals with moderate Alzheimer’s disease. Three aspects were evaluated: brain mapping with conventional tomography, metabolic changes in regional neurons evaluated with PET, so as cognitive function. Results showed entorhinal and hippocampal zones had modifications after stimulation. The amnesia was ameliorated, using a neuropsychological battery, posterior to modulation of these targets for 12 months (Laxton et al., 2010). Based on the findings, other researchers have followed this method, obtaining similar results to the ones first published (Fontaine et al., 2013; Sankar et al., 2015; Ponce et al., 2016; Leoutsakos et al., 2018; Germann et al., 2021; Targum et al., 2021; Barcia et al., 2022; Ríos et al., 2022).

Two more sites of stimulation to treat amnesia have been proposed: Nucleus Basalis Meynert (NBM) and Ventral Capsule/Ventral Striatum. Laxton and Lozano (2013) proposed the first target explaining that: “the study of dementia is preliminary and limited.” They showed evidence of the use of DBS in NBM for Alzheimer’s disease and dementia in Parkinson’s disease with initially hopeless results (Laxton and Lozano, 2013). Later on, Kuhn et al. (2015) implanted six patients with good responses and four of them with no side effects. In the last years, other studies reported positive results as well (Baldermann et al., 2018; Dürschmid et al., 2020; Jiang et al., 2022).

On the other side, the Ventral Capsule/Ventral Striatum stimulation produced modifications of the frontotemporal circuits involved in learning and memory formation. At this moment there is a lack of experience in this area (Scharre et al., 2018).

### Schizophrenia

Schizophrenia is an illness with controversial use of surgery, based on the molecular, genetic, and cellular changes with animal models (Heath, 1975; Ma and Leung, 2014; Schwabe and Krauss, 2018). The most important question is what part of the brain should be modified. It is necessary to known which circuits are involved in negative and positive symptoms.

The first attempt to use DBS in schizophrenia was performed by Heath, on patients with other comorbidities including sexual disturbances. Back then, they were different scientific and ethical views compared with today’s standards, so Dr. Heath was severely criticized (Heath, 1975).

**FIGURE 1:**
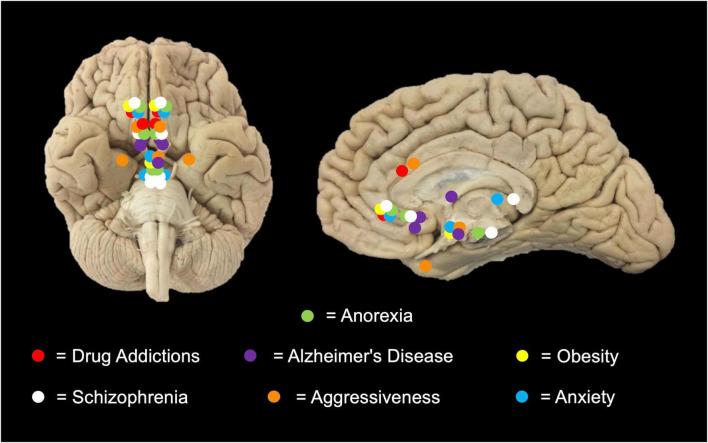
Targets of new indications for DBS. In this representative scheme, different anatomical DBS sites are shown according to pathologies: Addictions (Cingulum, Nucleus accumbens [Na]); Anorexia (Rostromedial Tegmental Nucleus, Subcallosal cingulate [Sc]); Anxiety (Na, Bed Nucleus of Stria Terminalis, Hypothalamus); Aggressiveness (Posterior hypothalamus, cingulum and amygdala); Alzheimer’s disease (Fornix/hypothalamus, Nucleus Basalis of Meynert; Ventral capsule/ventral Striatum); Schizophrenia (Na, anterior Corpus callosum, Habenula); and Obesity (Anteromedial Hypothalamus, Na) for performing DBS therapy.

Corripio et al. (2020) published the first paper with a stronger methodology to evaluate patients with schizophrenia. The article was focused on the Na or subgenual ACC bilateral neuromodulation (Corripio et al., 2020). They performed a randomized, double-blind crossover phase lasting 24 weeks, showing 25% of symptoms amelioration. Wang et al. (2020) performed DBS in the habenula in two patients, founding only a response in one of them, after 12 months of follow-up. Later, Corripio et al. (2022) described the possibility of two targets: the ventral tegmental area and the substantia nigra pars reticulata, but the experience at the moment is poor to get conclusions (see Figure 1).

### Neuroprosthetics or brain-machine interfaces in psychosurgery?

Another important aspect of this review is that DBS can be used to enhance human capacities and not just overcome psychiatric disorders, using electrophysiological theory and neuroprosthetics. It will be explained on the neurological, technical, philosophical, and ethical levels the implications of computer sciences with neurosciences.

BMIs, also known as brain-computer interfaces or neuroprosthetics, are devices that record signals from the nervous system and control external devices or hybrid systems to help restore communication or movement (Benabid et al., 1991; Albright et al., 2000; Hochberg et al., 2012). Neuroprosthetics have three parts: (1) a sensor that detects the electrical activity of the brain, (2) a signal processor that decodes the movement in the neural activity through algorithms, and (3) an effector that carries out the desired action using digital systems (i.e., cursor, computer), electromechanical prostheses and/or direct neuromuscular reactivation (Benabid et al., 1991; Raspopovic, 2021).

Nowadays, the use of BMI is emerging, and it is expected that it will eventually have a specific clinical application (Kansaku, 2021). For this purpose, closed-loop systems, fast and efficient algorithms, the development of better models of movement, sensory-motor feedback, miniaturized hardware, and wireless broadband systems are required (Ajiboye et al., 2017; Savage, 2018; Schwemmer et al., 2018; Simeral et al., 2021; Yadav et al., 2021). Findings such as the increment in spatial memory in epileptic patients with entorhinal electrical stimulation (Topalovic et al., 2020) have questioned the potential use of DBS and BMI in people with age-related cognitive deficits, and people without neurological pathologies (Suthana et al., 2012; Zimerman et al., 2013).

Neuroenhancement that uses chemicals, artifacts, or techniques on the brain to increase neurological capabilities—especially cognitive functions—is a new and complex issue with various positions (Hochberg et al., 2012; Suthana et al., 2012; Zimerman et al., 2013). Humans can receive diverse focal modifications in their bodies, which could be accumulative. In the long term, this could theoretically modify beings from current humans into transhumans. Even more, it is possible to use technology not only to extend human cognitive capabilities to a superhuman level, but also to transcend the biological limits of the mind and consciousness. The idea is to merge neural biological networks with artificial neural networks in order to end psychiatric diseases.

All of the aforementioned have profound ethical and philosophical issues that concern human survival as individuals and humanity (Koch, 2010; Zimerman et al., 2013; Clark and Parasuraman, 2014). A possible artificial selection of transhumans (with neuroprosthetics and/or genetic engineering), could lead to a hyperspecialized human race directed by them. Mental illnesses and limbic malfunctioning could be abolished completely. Psychiatric diseases will be a topic from the past, but mind afflictions such as biohacking might be a concern in these “new neural networks composed of cells and inorganic technology” (Yetisen, 2018).

On the other hand, the biological transcendence of consciousness upgraded to digital systems implies virtually non-mortal entities, a situation that could break the *status quo* and stagnation of human civilizations (Hochberg et al., 2012; Raspopovic, 2021). Undoubtedly the horizons of neuroenhancement are far and surely deep, speculation about issues allows us to imagine some possible contexts, but it is in our hands to define the results. It is important to discuss these topics from now on from different perspectives, including psychiatric diseases and their obliteration: it is important to understand what the concept attributed to the person means, and what is the exact role of neuroprosthetics in the essence of the human being (Carrillo-Ruiz et al., 2020, 2022).

## Conclusion

Limbic surgery has new indications to treat anxiety, eating disorders, addictions, aggressiveness, schizophrenia, and Alzheimer’s disease. It is important to take into consideration the anatomical sites of DBS use, as well ethical and scientific committees should be present when approaching each patient. Transhumanism is a contemporary option for DBS use in treating or enhancing some cognitive abilities, and a possible technology to abolish psychiatric diseases in the future.

## Author contributions

JC-R originated the idea and abstract. JC-M made the figure. JB created the table. FJ-P and LG-M contributed with psychosurgery indications in the manuscript. JN-O checked anatomical functional details as well as correcting style. RM-F added information regarding neuroprosthetics, neuroenhancement, and transhumanism. FV reviewed the text and contributed with expertise in the field. All authors participated in the creation of this manuscript by writing evenly.
